# Epithelioid sarcoma in the chest wall: a case report and literature review

**DOI:** 10.1186/s40792-018-0483-7

**Published:** 2018-07-13

**Authors:** Teiko Sakurai, Hidenori Kusumoto, Tomoko Wakasa, Yoshio Ohta, Eiichi Konishi, Hiroyuki Shiono

**Affiliations:** 1 Department of General Thoracic Surgery, Kindai University Nara Hospital, Otoda-cho 1248-1, Ikoma, Nara 630-0293 Japan; 2 Diagnostic Pathology and Laboratory Medicine, Kindai University Nara Hospital, Otoda-cho 1248-1, Ikoma, Nara 630-0293 Japan; 3 Department of Surgical Pathology, Kyoto Prefectural University of Medicine, Kajii-cho 465, Kawaramachi-Hirokoji, Kamigyo-ku, Kyoto, 602-8566 Japan

**Keywords:** Proximal-type epithelioid sarcoma, Chest wall, Wide resection, Polytetrafluoroethylene, Reconstruction

## Abstract

**Background:**

Epithelioid sarcoma (ES) is a rare variant of soft tissue sarcoma. The proximal type of ES occurs in various locations. We present a resected case with proximal-type ES that occurred in the chest wall and discuss the relevant literature.

**Case presentation:**

A 47-year-old woman was referred for a 6-month history of a right anterior chest mass with tenderness. Chest computed tomography showed an invasive chest wall mass with calcification surrounding the third rib. Aspiration biopsy cytology suggested malignancy. We performed wide resection, including the middle part of the pectoralis major muscle, the pectoralis minor muscle, the third and fourth ribs, and reconstruction of the chest wall, using a 2-mm polytetrafluoroethylene patch. Severe deformation of the chest wall was avoided. Postoperative physical therapy of the shoulder was effective for the continuous pain and weakness of the arm. She has remained alive for 1 year and 10 months without recurrence. Our literature review showed five previously reported cases of ES in the chest wall, and all of these were surgically resected. Two of these patients suffered from frequent local recurrence and died of disease.

**Conclusions:**

ES in the chest wall is rare. Previous reports have indicated that surgical resection with tumor-free margins is essential for treatment. We performed complete resection of the tumor in our case, and a polytetrafluoroethylene patch was effective for reconstructing the deficit in the chest wall.

## Background

Epithelioid sarcoma (ES) was first described by Enzinger in 1970 [1]. ES represents less than 1.0% of all sarcomas [2]. Two subtypes of ES are currently recognized, including (i) the conventional/ classic type and (ii) the proximal type. The proximal type of ES occurs in various locations, such as truncal tissue, and the buttocks, thighs, head, and neck [3].

## Case presentation

**Fig. 1:**
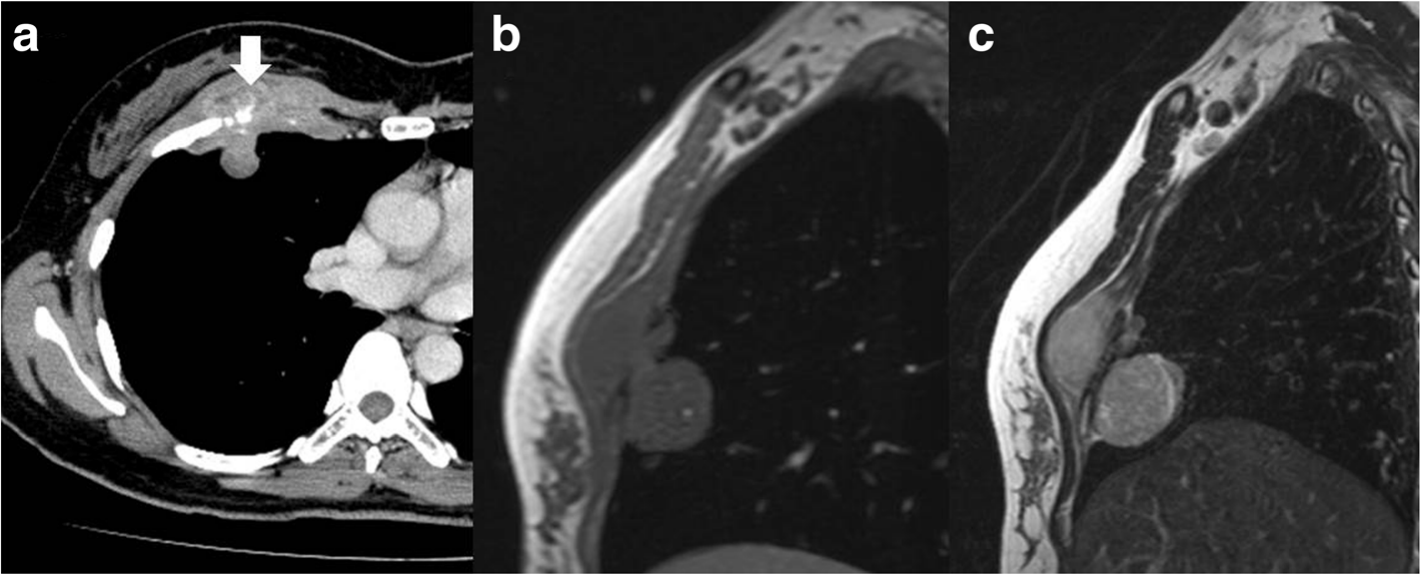
Enhanced chest CT shows a right chest wall tumor with calcification (white arrow) (**a**). Chest MRI shows an invasive dumbbell-shaped tumor, which is isointense on T1-weighted images (**b**) and heterogeneously hyperintense on T2-weighted images (**c**)

A 47-year-old woman was referred with a 6-month history of a right anterior chest mass. A physical examination showed a palpable firm mass with tenderness in the right anterior chest. Her routine laboratory investigations were within the biological reference range. Enhanced chest computed tomography (CT) showed a dumbbell-shaped mass with calcification, and its anterior portion was located under the pectoralis minor muscle and the posterior portion projected to the thoracic cavity (Fig. 1a). Chest magnetic resonance imaging (MRI) showed an invasive tumor, which was isointense on T1-weighted images and heterogeneously hyperintense on T2-weighted images (Fig. 1b, c). Aspiration biopsy cytology performed by a previous physician had shown malignancy, and no evidence of distant metastasis was found. Therefore, we planned surgical resection of the tumor with chest reconstruction. The patient was placed in the supine position. We first examined inside the thoracic cavity with thoracoscopy through the seventh intercostal space and found no lung invasion of the tumor. Wide resection, including the middle part of the pectoralis major muscle, the pectoralis minor muscle, and the third and fourth ribs, was performed. A negative margin of the tumor was identified by frozen sections. We used a 2-mm expanded polytetrafluoroethylene (ePTFE) patch (Gore Dualmesh; W.L. Gore & Associates, Flagstaff, AZ, USA) for chest wall reconstruction and covered it with spared skin and breast (Fig. 4). The operation time was 3 h and 33 min, and intraoperative blood loss was 64 ml.

**Fig. 2:**
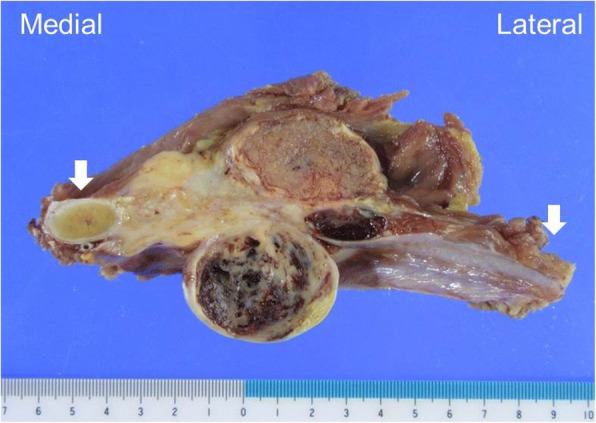
The resected specimen shows a tumor surrounding the right third rib (white arrow)

**Fig. 3:**
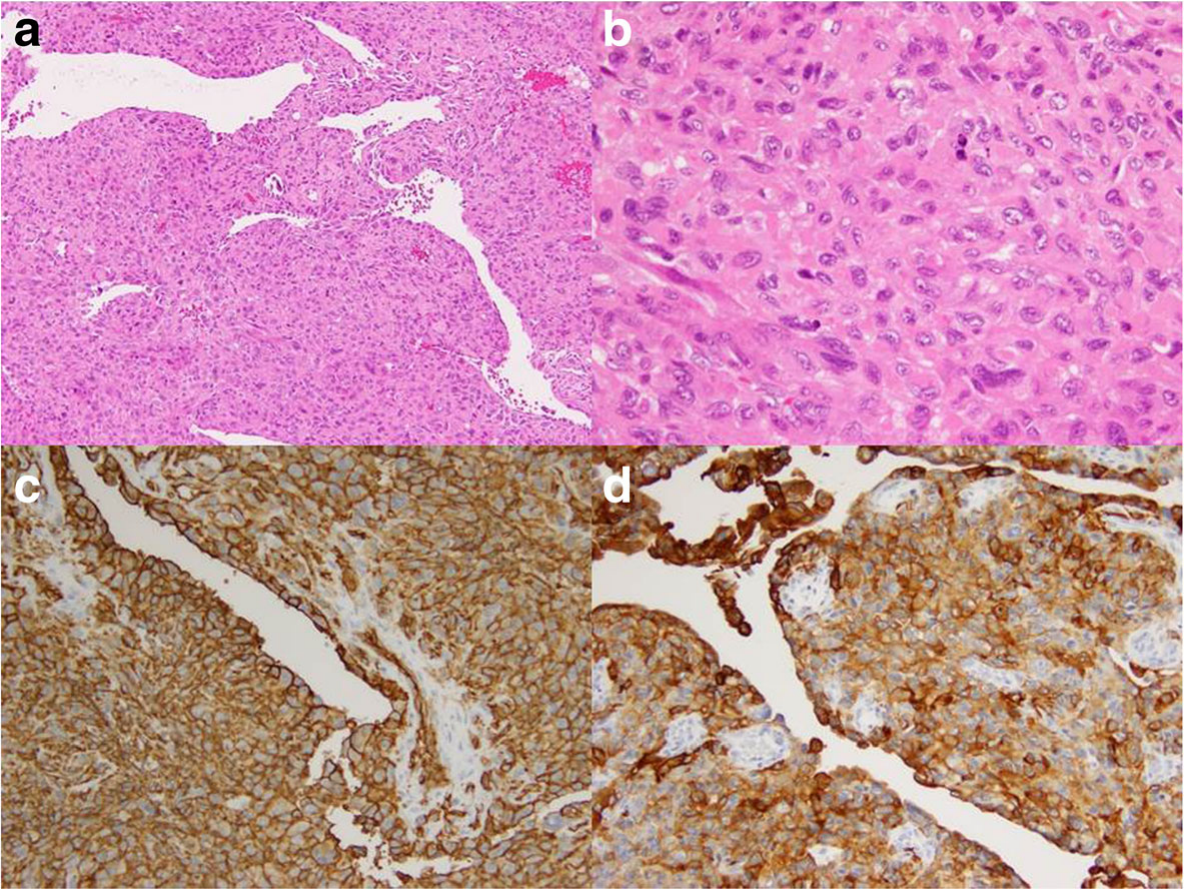
Tumor cells are epithelioid and arranged in a large sheet-like pattern with microcalcification in hematoxylin and eosin staining (**a**, **b**). CD34 is strongly and diffusely expressed in the cytomembrane (**c**) and cytokeratin (AE1/AE3) is also expressed in the cytoplasm of most tumor cells (**d**)

The resected specimen was a firm tumor that surrounded the third rib (7.5 cm) (Fig. 2). Microscopically, the tumor cells showed an epithelioid appearance with cytoplasmic eosinophilia. The epithelioid cells had large vesicular nuclei and were arranged in sheet-like pattern. In some locations, scattered microcalcification was observed (Fig. 3a, b). Immunohistochemical staining showed expression of CD34 and cytokeratin (AE1/AE3) (Fig. 3c, d), but no expression of CD31, Sox10, Stat6, and integrase interactor 1 (INI1) in tumor cells. The diagnosis was proximal-type ES in the right chest wall. The French Fédération Nationale des Centres de Lutte Contre Le Cancer grading system was grade 2.

We started physical therapy on postoperative day 6 for local pain and limitation of shoulder motion. These symptoms improved by physical therapy, and she was discharged on postoperative day 18. Although weakness of the arm and chronic pain had been persistent, she could be reinstated in former factory work. We did not perform postoperative adjuvant chemotherapy or radiation therapy. She has remained alive for 1 year and 10 months without recurrence.

## Discussion and conclusions

Primary chest wall sarcomas are rare in all malignant neoplasms. More than half of malignant tumors of the chest wall are metastatic lesions from distant organs or invasion from contiguous structures [4]. In a large series of patients with soft tissue sarcoma without metastases, tumors of only 3.8% of patients were located in the chest wall [5, 6]. The chest wall is also a well-known site of radiation-induced sarcomas [7].

For diagnosis of ES, any imaging system is not helpful because CT or MRI findings are nonspecific [8]. Proximal-type ES tends to affect the older population and has a worse prognosis than the conventional/classic type [9, 10]. Surgical resection with a tumor-free margin is essential in malignant soft tissue tumors, including ES, to prevent recurrence [2]. The effect of adjuvant therapy remains unclear [11, 12]. ES in the chest wall is rare. A literature review showed five cases of ES in the chest wall, including the present case (Table 1) [10, 13, 14]. All cases were reported from Asia, and none of them were from other areas [9, 15]. The age range of these cases was from 24 to 64 years old. There was one case with local metastasis, and none with distant metastasis at presentation. All of these cases underwent surgical resection. Two of five patients suffered from multiple times of local recurrence and finally died of disease. Although adjuvant therapy was performed in two cases, the effect was not clearly described. The present case was also followed up without any adjuvant therapy.

**Table 1:** Previous reports of ES in the chest wall

First author (country)	Age (year)	Sex	Size (cm)	Metastasis at presentation	Excision	Adjuvant therapy	Resection for local recurrence (times)	Outcome (months)
Hasegawa [13] (Japan)	48	F	6.0	No	Marginal resection	RT	2	DOD (132)
Aizawa [14] (Japan)	64	M	5.5	No	Wide resection	No	3	DOD (22)
	24	F	2.0	Local	Wide resection	No	0	NED (16)
Rekhi [10] (India)	58	M	4.0	No	Wide resection	CT and RT	NK	LOF (NK)
Present case (Japan)	47	F	7.5	No	Wide resection	No	0	NED (20)

*RT* radiation therapy, *CT* chemotherapy, *NK* not known, *DOD* died of disease, *NED* no evidence of disease, *LOF* lost to follow-up

ES is defined as a malignant mesenchymal neoplasm that exhibits epithelioid cytomorphology and is predominantly an epithelioid phenotype. Pathologically, ES characteristically shows diffuse expression of epithelial membrane antigen and cytokeratins, which is similar to metastatic carcinoma. Therefore, ES (especially the proximal type) is often confused with metastatic carcinomas [3]. However, CD34 is expressed in half of ES cases and INI1 (also known as hSNF5, SMARCB1, and BAF47) is deficient in approximately 90% of ES cases in contrast to metastatic carcinoma [3, 13, 16, 17]. INI1-deficient tumors have also been reported, such as rhabdoid tumor of the kidney, renal medullary carcinoma, epithelioid malignant peripheral nerve sheath tumor, myoepithelial carcinoma, and extraskeletal myxoid chondrosarcoma [18]. In the present case, no expression of CD31, Sox10, and Stat6 contradict the diagnosis of angiogenic, neurogenic, or solitary fibrous tumor [19–21]. Therefore, these immunohistochemical stains were helpful for diagnosing ES.

**Fig. 4:**
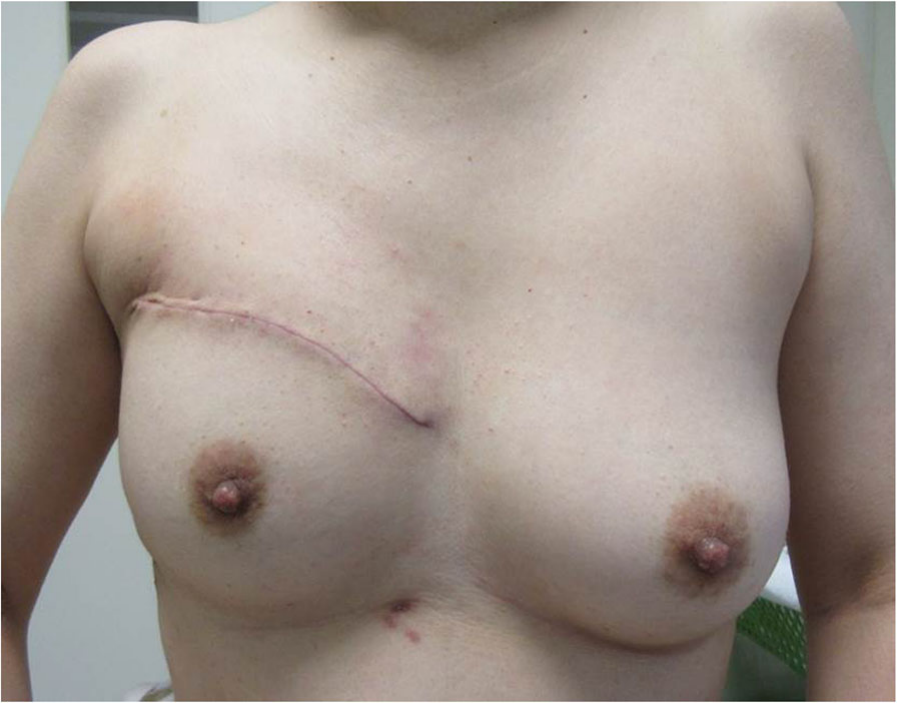
The deficit of muscles was covered with the spared skin and breast, and severe deformation was avoided

The goals of reconstruction of the chest wall are adequate stability in respiration (prevention of paradoxical movement), water- and airtight closure, and an acceptable cosmetic appearance [22]. The use of alloplastic and/or xenogenic materials, as well as muscle flap repair, is well established [23–26]. However, Dingemann and colleagues reported one of eight pediatric cases suffered from rigid prosthetic material dislocation as a long-term complication in surgical reconstruction of the chest wall [27]. We used a 2-mm ePTFE patch for stabilization of the chest wall because it enabled us to reconstruct the chest wall safely and easily [27, 28]. Severe deformation was avoided by covering with the pared skin and breast (Fig. 4). However, loss of the middle part of the pectoralis major muscle induced continuous weakness in adduction, horizontal flexion, and internal rotation of the arm. Stretching and training of residual muscles may improve regional pain and prevent contracture of the shoulder.

In conclusion, we describe the clinical course and surgical treatment of a patient presenting with ES in the chest wall, which is a rare condition.

## Abbreviations

CT: Computed tomography
ePTFE: Expanded polytetrafluoroethylene
ES: Epithelioid sarcoma
INI1: Integrase interactor 1
MRI: Magnetic resonance imaging
